# High-dose vitamin D therapy and prolonged partial remission of type 1 diabetes — a case report

**DOI:** 10.3389/fendo.2026.1788049

**Published:** 2026-03-31

**Authors:** Adriana Chader-Gata Garcia, Kristina Cossen, Benjamin Udoka Nwosu

**Affiliations:** 1 Division of Endocrinology, Department of Pediatrics, Cohen Children’s Medical Center, Hofstra/Northwell, Queens, NY, United States; 2 Division of Pediatric Endocrinology, Children’s Healthcare of Atlanta, Atlanta, GA, United States; 3 Emory University School of Medicine, Atlanta, GA, United States; 4 Donald and Barbara Zucker School of Medicine at Hofstra/Northwell, Hempstead, NY, United States

**Keywords:** adolescents, children, honeymoon phase, partial clinical remission, type 1 diabetes, vitamin D

## Abstract

Clinical trials demonstrating β-cell protection by high-dose vitamin D supplementation have led to the adoption of adjunctive vitamin D therapy in stage 3 type 1 diabetes (T1D). We present the case of a 12-year-old male child who received high-dose vitamin D therapy from 1–27 months following his diagnosis with T1D. His serum 25-hydroxyvitamin D rose from 25 ng/mL to 65 ng/dL within 3 months and remained >40 ng/mL. Hemoglobin A1c (A1c) decreased from 13.8% at diagnosis to persistently <7%. Time in range remained >70% for 24 months, and insulin-dose adjusted A1c (IDAA1c), the clinical marker of partial clinical remission, decreased from 17 to <9 for the duration of therapy. This description of persistence of partial clinical remission (PR), defined as IDAA1c ≤9, for 27 months in a child receiving adjunctive high-dose vitamin D therapy is a build up from our randomized controlled trial of high-dose vitamin D where we demonstrated significant blunting of temporal rise in A1c and IDAA1c over a 12-month period. This prolonged remission by high-dose vitamin D is clinically significant as it positions vitamin D as an affordable therapeutic agent to prolong PR and reduce the short- and long-term complications of T1D. Long-term studies are needed to determine the ultimate duration of these beneficial effects.

## Introduction

The publication of a randomized controlled trial (RCT) (1, 2) of high-dose vitamin D in children with newly diagnosed type 1 diabetes (T1D) and a protocol (3) for its implementation has led to the adoption of adjunctive vitamin D therapy in various centers in the United States and abroad. T1D is a chronic disease resulting from autoimmune destruction of the pancreatic β-cells leading to insulinopenia and persistent hyperglycemia (4, 5). A “honeymoon” phase, also known as partial clinical remission (PR), may follow the diagnosis of overt T1D. PR is marked by an increased functionality of the surviving β-cells with associated endogenous insulin and C-peptide production (6, 7). PR typically lasts for 3–12 months (8), but could extend for decades into the established phase of T1D (9). Data from the landmark Diabetes Control and Complications Trial showed that prolonging the PR reduced both the short- and long-term complications of T1D (10). Consequently, decades of clinical trials have sought to determine the best approaches to prolong PR without significant adverse effects (4, 11, 12). We recently published a 12-month randomized controlled trial of high-dose oral vitamin D_2_ (ergocalciferol), 50,000 international units per week for 2 months, and then biweekly for 10 months versus placebo to protect residual β-cell function (RBCF) and prolong PR in children and adolescents, ages 10–21 years, with new onset T1D (13). The trial showed that ergocalciferol significantly decreased fasting proinsulin to C-peptide (PI:C) ratio, the percent change from baseline in the area-under-the-curve (%ΔAUC) of C-peptide, tumor necrosis factor-alpha (TNF-α) concentrations, and the temporal trends in both hemoglobin A1c (A1c) and the insulin-dose adjusted A1c (IDAA1c) levels, a marker of residual β-cell function. These positive results were associated with no vitamin D related adverse effects. As a result of these safety and efficacy data, endocrinologists now introduce adjunctive vitamin D therapy to their patients with new-onset T1D. We present the history and early therapy of a male child who received high-dose vitamin D supplementation from 1–27 months following his diagnosis with T1D and remained in PR throughout the period of treatment. He received vitamin D_2_, ergocalciferol, 50,000 international units (i.e., one capsule) at home, by mouth each week for 4 weeks, and then once every other week for the subsequent 26 months.

## Case presentation

### Ethical and legal declarations

The patient’s data were de-identified and anonymized prior to presentation in this manuscript in compliance with the Declaration of Helsinki.

A 12-year-old African American male child presented with symptoms of polyuria and polydipsia at a tertiary hospital in stable condition. His physical examination was normal except for mild dehydration. He measured 151.5 cm (48.8 percentile) in height, and weighed 37.1 kg (23.4 percentile), with a BMI of 16.2 kg/m^2^ (17.01 percentile). His initial laboratory tests showed a random blood glucose of 175 mg/dL, A1c of 13.8% [normal 4.4-5.6%], venous pH of 7.28 [normal 7.35-7.45], bicarbonate of 14 mEq/L [normal 20–28 mEq/L], and an anion gap of 22.8 mmol/L [normal 2–12 mEq/L]. His diabetes-associated autoantibody profile showed an elevated glutamic acid decarboxylase (GAD) of 124.9 IU/mL [normal range <5 IU/mL], and undetectable islet antigen 2 (IA-2) of <5.4 units/mL [positive value is 7.5 units/mL], and insulin autoantibodies (IAA) of <0.4 uU/mL. He was diagnosed with T1D based on his clinical presentation and laboratory data. He received intravenous fluid therapy and insulin to correct his dehydration and hyperglycemia, respectively. He was subsequently transitioned to subcutaneous insulin based on basal-bolus regimen. He was started on a continuous glucose monitor (CGM) during his admission and was discharged home on the second day of admission. Four weeks after his diagnosis, he was started on home-administered, high-dose vitamin D supplementation using oral vitamin D_2_, ergocalciferol capsules at a dose of 50,000 international units (i.e. one capsule by mouth) per week for the first 4 weeks, and then every other week for the subsequent 26 months according to Nwosu’s protocol for high-dose vitamin D supplementation in children with T1D (3). He received the 50,000 international units of ergocalciferol orally at home (not in the hospital) after a meal, however, on some occasion he took the capsule with his meal or before a meal. At each routine 3-month visit, his endocrinologist performed a detailed physical examination, reviewed his anthropometric measures, point-of-care A1c, total daily dose of insulin (TDDI), CGM metrics, and measured 25-hydroxyvitamin D level. Ten months after his diagnosis, he was transitioned to continuous subcutaneous insulin infusion (CSII) via an insulin pump that was integrated with his CGM forming an automated insulin delivery (AID) system with an in-built algorithm. His TDDI was initially estimated based on his basal insulin dose and his insulin to carbohydrate ratio together with average carbohydrates consumed while he was on multiple daily injections. When he transitioned to CSII, his TDDI was obtained from his insulin pump reports. The patient’s partial clinical remission (PR) status was determined using the clinical marker of PR, the insulin-dose-adjusted A1c (IDAA1c), which is calculated with the formula: A1c (%) + 4 x TDDI (units/kg/day). An IDAA1c value of ≤ 9 is consistent with PR (8).

**Figure 1:**
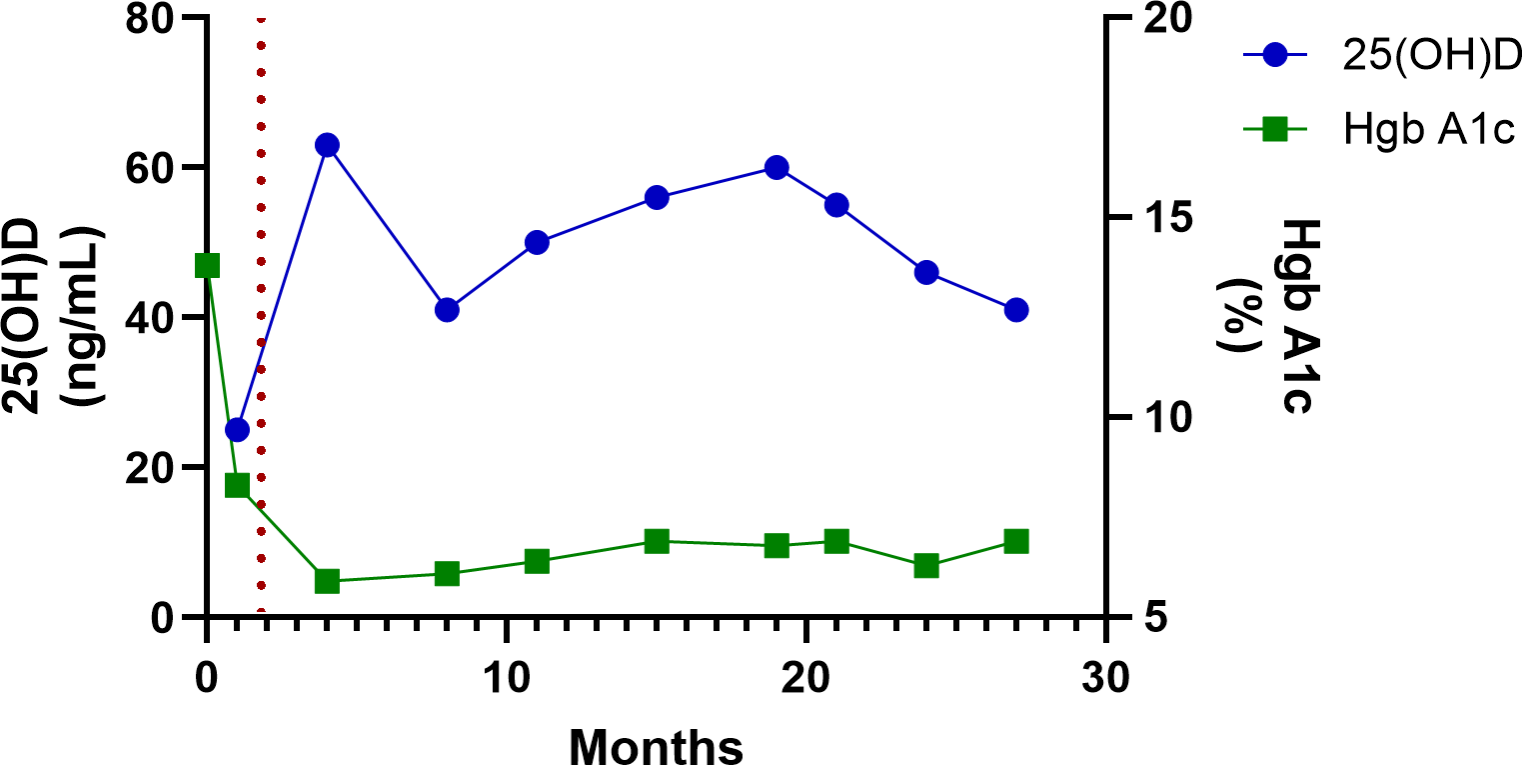
A graphical representation of the temporal relationship between serum 25-hydroxyvitamin D concentration and A1c during routine 3-month clinic visits for diabetes care for 27 months. The red dotted line indicates the time of initiation of high-dose vitamin D therapy.

**Figure 2:**
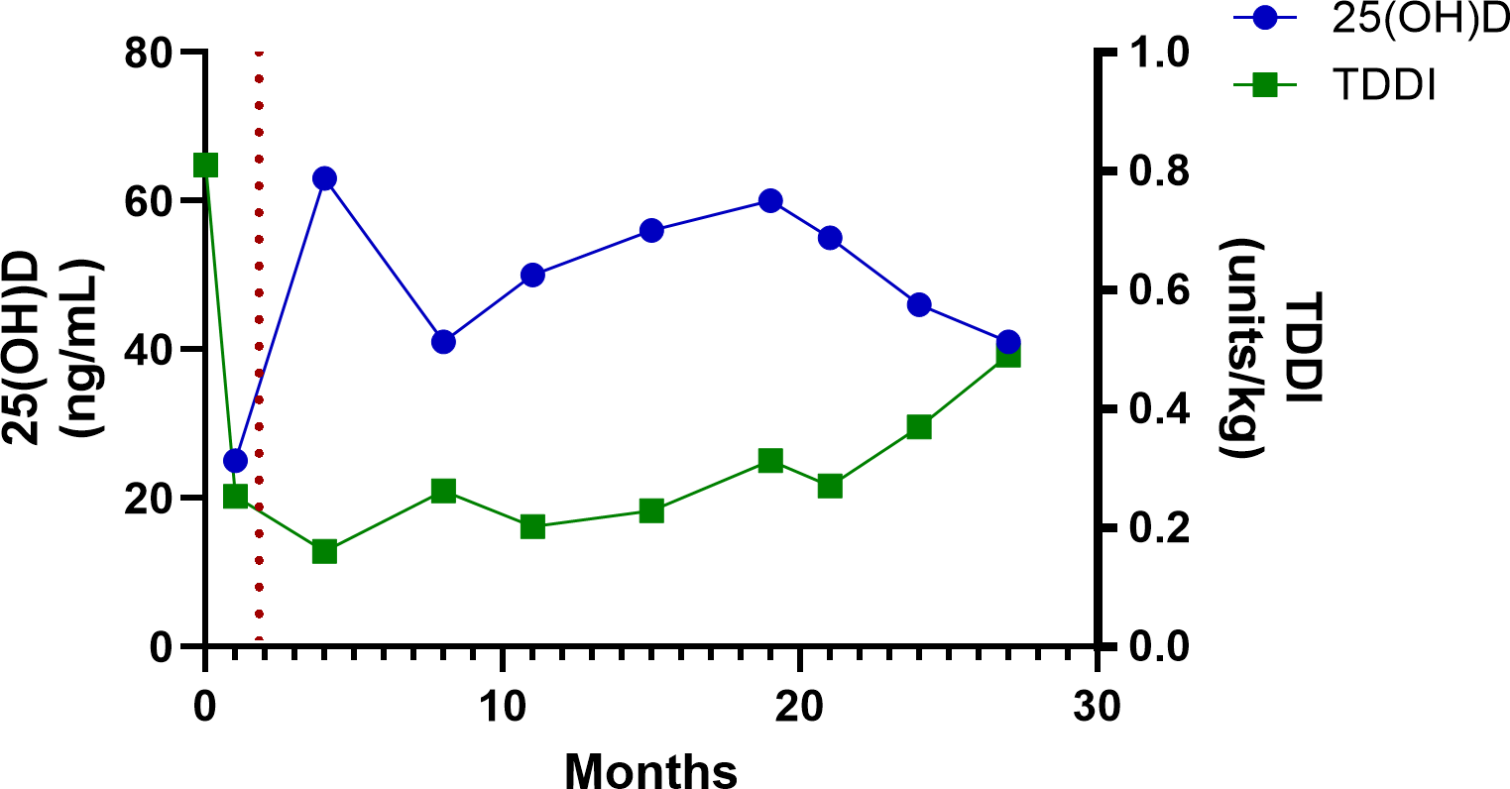
A graphical representation of the temporal relationship between serum 25-hydroxyvitamin D concentration and total daily dose of insulin during routine 3-month clinic visits for diabetes care for 27 months. The red dotted line indicates the time of initiation of high-dose vitamin D therapy.

**Figure 3:**
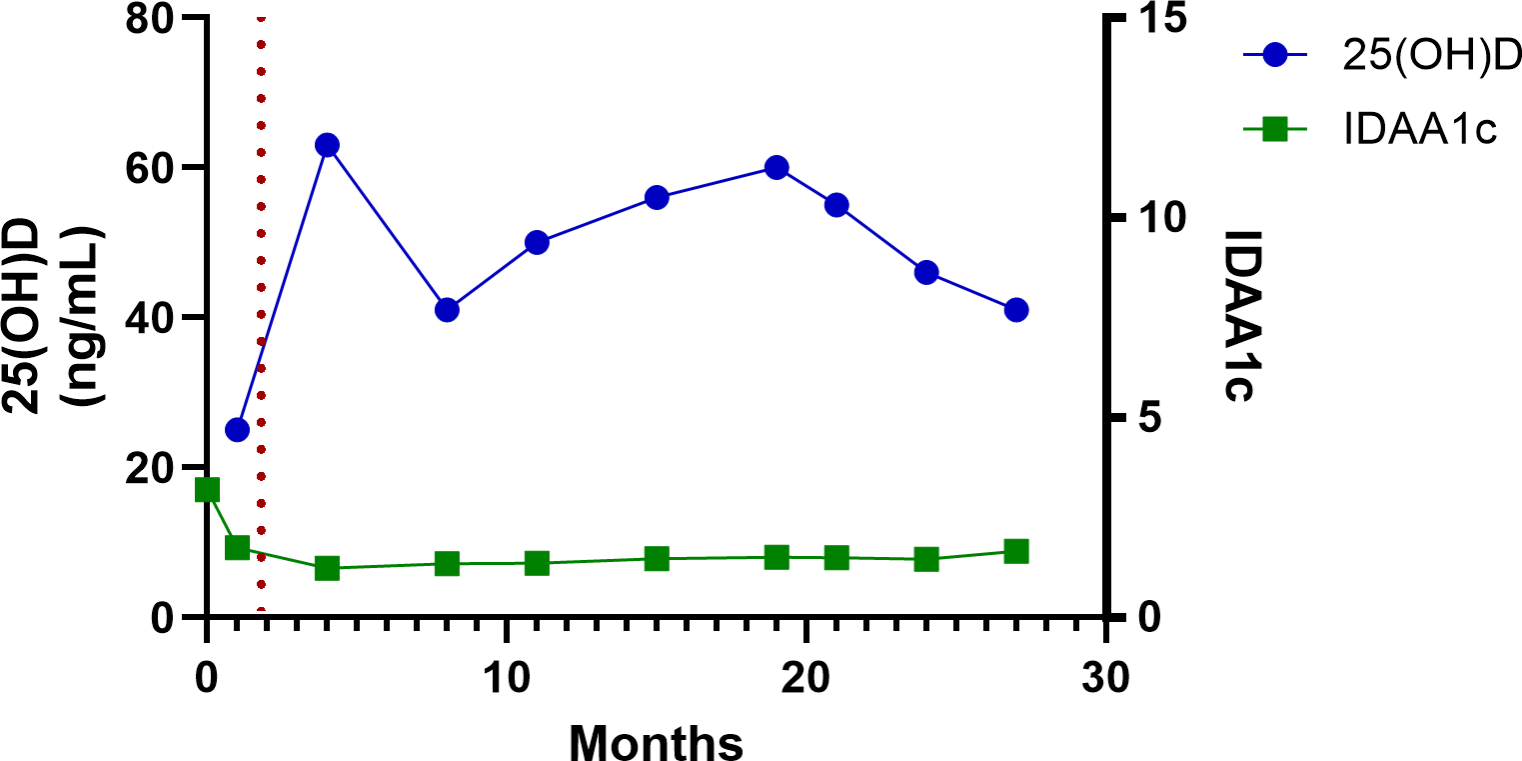
A graphical representation of the temporal relationship between serum 25-hydroxyvitamin D concentration and insulin-dose-adjusted A1c during routine 3-month clinic visits for diabetes care for 27 months. The red dotted line indicates the time of initiation of high-dose vitamin D therapy.

The patient’s baseline serum 25-hydroxyvitamin D [25(OH)D] concentration, drawn 4 weeks after his diagnosis with T1D, but before his treatment with high-dose vitamin D, was 25 ng/mL. After starting high-dose vitamin D therapy, serum 25(OH)D rose to 63 ng/mL within 3 months and remained >40 ng/mL for the remainder of the period of observation (**Table 1**; **Figures 1**–**3****).** Hemoglobin A1c decreased from an initial value of 13.8% at diagnosis to 5.9% at the 4^th^ month and persisted at <7% for the remainder of the period of observation (**Table 1**; **Figure 1**). The CGM metrics showed an average blood glucose range of 123–162 mg/dL, time in range (70-180mg/dL) of >70% for 24 months, and 69% at the 27^th^ months (**Table 1**). His time below range (<70 mg/dL) was <1% throughout the period of observation (data not shown). The analysis of insulin requirements showed that the TDDI decreased from a high value of 0.81 units/kg/day at diagnosis to 0.25 units/kg/day in the first month, with a further decline to 0.16 units/kg/day in the 3^rd^ month. TDDI remained stable in the low range for age and pubertal status, at <0.5 units/kg/day, throughout the period of observation (**Table 1**; **Figure 2**). The analysis of the changes in the marker of PR, the IDAA1c, showed that IDAA1c decreased from a high of 17 (consistent with an absence of partial remission) in the first month, to a normal value of 6.5 (normal range is <9) at the 4th month following the diagnosis of T1D, and remained at <9 for the duration of observation (**Table 1**; **Figure 3**). The timing of the normalization of IDAA1c to <9 coincided with the elevation of 25(OH)D to 63 ng/mL (**Figure 3**).

**Table 1:** Longitudinal parameters at baseline and during high-dose vitamin D supplementation.

Time from diagnosis (months)	Height (cm)	Height (%)	Weight (kg)	Weight (%)	BMI (kg/m^2^)	BMI (%)	A1c (%)	TDDI (units/kg)	IDAA1c	AVG glucose (mg/dL)	TIR (%)	25(OH)D (ng/mL)
0	151.5	48.83	37.1	23.37	16.16	17.01	13.8	0.81	17.03	–	–	–
1	151.3	43.52	39.5	32.37	17.26	34.68	8.3	0.25	9.31	135	88	25
4	152.7	40.26	37.4	17.14	16.04	12.20	5.9	0.16	6.54	123	95	63
8	153.6	36.18	38.2	15.70	16.02	10.45	6.1	0.26	7.15	141	81	41
11	155.8	34.09	39.1	13.99	16.11	9.31	6.4	0.20	7.21	128	87	50
15	157.5	31.08	41.6	17.67	16.77	15.32	6.9	0.23	7.81	134	87	56
19	158.9	26.26	44.4	22.07	17.58	24.50	6.8	0.31	8.05	151	76	60
21	160.8	29.00	43.0	14.00	16.63	10.00	6.9	0.27	7.96	133	86	55
24	161.5	26	44.6	16	17.10	14	6.3	0.37	7.78	155	75	46
27	164	30	48.1	24	17.88	23	6.9	0.49	8.86	162	69	41

25(OH)D, 25-hydroxyvitamin D; AVG, average; BMI, body mass index; A1c, hemoglobin A1c; IDAA1c, insulin-dose adjusted A1c; TDDI, total daily dose of insulin; TIR, time in range of glycemia.

## Discussion

We report a 12-year-old African American male child who received high-dose vitamin D therapy from 1–27 months following his diagnosis with T1D. The results support the hypothesis that high-dose vitamin D prolongs PR and improves metabolic or glycemic endpoints with no associated side effects or vitamin D toxicity (1, 2). The rationale for the vitamin D dosing regimen of 50,000 IU weekly for one month and then once every two weeks, in this case, was to raise serum 25-hydroxyvitamin D levels to the optimal range for the extra-skeletal functions of vitamin D, and then to maintain the level using once every other week dosing schedule (1). This real-world case report is consistent with a recent RCT which showed that high-dose vitamin D therapy protects the β-cells of the pancreas and reduces the temporal trends in both A1c and IDAA1c (1, 2). The RCT further showed that the mechanisms of vitamin D’s action include reductions in β-cell endoplasmic reticulum stress and systemic inflammation as shown by significant reductions in the PI:C ratio and serum concentrations of TNF- α respectively (13). Data from other supporting studies show that vitamin D supplementation could prolong PR by improving pancreatic β-cell survival and function through its immunomodulatory functions (14). Additional studies indicate that vitamin D supplementation is associated with reduced glycemia, increased C-peptide secretion, and decreased insulin requirements in individuals with T1D (15–17). In contrast, a systematic review (18) of the effect of vitamin D supplementation on glycemic control in children and adolescents with T1D which evaluated 10 clinical studies reported no consistent evidence on the effect of vitamin D supplementation on glycemia in youth with T1D. A closer look at this systematic review showed that though the included studies mostly used cholecalciferol as their intervention strategy, there was a significant variation in the dose and duration of vitamin D supplementation. Despite these inconsistencies in the dose of vitamin D and the duration of therapy, the systematic review found a significant reduction in glycemia in 50% of the studies when A1c was used as a marker of glycemic control. This suggests that the impact of vitamin D supplementation on glycemia is best elicited under a protocol of high-dose vitamin D supplementation over a prolonged period (1, 19). Another systematic review of the impact of vitamin D on glycemic control in children and adults with T1D that included six studies reported favorable reductions in A1c in subjects with newly diagnosed T1D who had significant vitamin D deficiency as depicted in three of the six included studies (20–22). However, this review found no overall evidence for significant reduction in A1c in the analysis of the six studies because of the high heterogeneity (I^2^ = 98.07%) in their analysis which suggested underlying variability in study population, intervention regimens, duration of follow up and disease stage, i.e., newly diagnosed versus established T1D. Thus, in summary, some of the negative and inconclusive results from studies on vitamin D supplementation in subjects with T1D stem from poor study design, which should not confound the positive results from well-designed studies.

It is important to discuss some potential improvements in pancreatic residual β-cell function (RBCF) with intensive insulin therapy, even before the initiation of insulin pump therapy. Data from the landmark Diabetes Control and Complications Trial (10) and some recent studies (23) suggest that intensive glycemic control could preserve RBCF (10, 24). However, other recent studies (25) and systematic reviews (26) failed to demonstrate that improved glycemia prolongs RBCF in subjects with new-onset T1D. A particularly interesting study (27) reported that RBCF at 2 years following a diagnosis of T1D was associated with the initial A1c and C-peptide concentrations at the time of diagnosis, and independent of initial insulin regimens. This suggests that improvements in glycemia by intensive insulin regimen may only have a marginal impact on RBCF (25).

It is also important to discuss our findings with respect to previous reports in non-White children and adolescents with T1D, who may have different characteristics from White children with T1D, but are often not well represented in mainstream discussion of the therapeutic impact of vitamin D in T1D. One of these was a 3-month open label trial (28) of oral vitamin D_3_ in 115 vitamin D-deficient children and adolescents of ages 1–19 years that was conducted from 2019–2022 in Sudan. The subjects received daily oral cholecalciferol as follows: 1–3 years, 2,500 IU daily; 4–8 years, 3000 IU daily; and 9–18 years, 4000 IU daily. The investigators found that vitamin D supplementation led to a significant increase in serum 25-hydroxyvitamin D; and decreased A1c in 67.7% of the subjects, though this did not reach statistical significance. They also reported a significant decrease in fasting blood glucose levels; and a non-significant change in insulin doses, though 25% of participants lowered their insulin doses. A retrospective, cross-sectional study (29) that compared the demographic and clinical characteristics of children from ethnic minorities and non-Hispanic white children with newly diagnosed T1D reported that Hispanic children, but not African American children, had higher random serum C-peptide concentrations after adjusting for confounders. This study was limited by its retrospective cross-sectional design, the use of non-standardized random C-peptide samples, and a relatively small number of minority children in the study. Another study (30) published in 2018 reported a significantly lower rate of partial clinical remission (PR) in African Americans (AA) compared to Non-Hispanic Whites (NHWs) in the first 3 years of diagnosis with T1D, while detecting no differences in lipid profile between the AA and NHW groups. However, in a letter to the editor, Nwosu (30) pointed out that the detection of lower rates of remission in African American children in the first 3 years of diagnosis of T1D, without evidence of early phase dyslipidemia (31), suggested that the finding on differences in remission frequency was misleading. This was because the insulin-dose adjusted A1c (IDAA1c) formula, which was used to calculate PR in the cohort, was generated using data from European and Japanese youth with T1D (8), and therefore, was not validated in ethnic minorities in the United States who possess unique characteristics that differ from the European and Japanese T1D cohorts that were used to derive the IDAA1c formula. Compared to their European and Japanese counterparts, US minority youth with T1D have higher BMI values (8, 30); and higher mean HbA1c value of 0.4% for the same mean glucose concentration (32). Therefore, IDAA1c easily underestimates the frequency of PR in minority children with T1D. Thus, conclusions on the phenotype and early biochemical and prognostic profiles of minority children with new onset T1D should be based on validated markers for this population, and well-designed randomized controlled trials with an adequate sample size.

Some of the reasons for publishing this case of long-term, high-dose vitamin D therapy in a child include the sustained impact of high-dose adjunctive vitamin D therapy on core clinical endpoints, vitamin D’s safety profile, and its ease of administration. We previously demonstrated a sustained impact of high-dose vitamin D therapy on PR and metabolic endpoints in a 12-month RCT (1). In that study, the impact of vitamin D on residual β-cell function, marked by %change from baseline in the C-peptide area-under-the-curve, appeared to strengthen with time (13), suggesting a prolongation of PR. The boy in this case report showed an ongoing PR status, marked by an IDAA1c of <9, at 27 months from T1D diagnosis, with no indication of attenuation of PR (**Figure 3**). This suggests that this patient’s PR could last for several more years. Additionally, this patient showed a favorable metabolic profile marked by a sustained decrease in A1c (**Figure 1**) and TDDI (**Figure 2**). Furthermore, the safety of long-term, high-dose vitamin D supplementation as demonstrated in this patient is reassuring and important for patients, parents, and endocrinologists who wish to start their children or patients on high-dose vitamin D therapy. Finally, this report shows that it is practicable to prolong PR using an affordable and easily accessible agent, vitamin D, without health insurance approval or being priced out of therapy by exorbitant drug costs.

The major limitation of this report is that the data were derived from a single patient, and so causality cannot be inferred. However, the achievement of this degree of superior glycemic endpoints and prolonged PR in an African American child is remarkable. This is because minority children with T1D in the United States often have suboptimal glycemia compared to their white peers and often lag behind their white peers in accessing diabetes technology. The observation that improvements in glycemia and PR occurred during vitamin D therapy, and before the initiation of pump therapy, is significant and excludes the use of automated insulin delivery system as the primary reason for the positive outcomes.

## Conclusion

The combination of published data from a recent RCT on high-dose vitamin D therapy in T1D and this real-world case report from an independent tertiary institution, suggest that high-dose vitamin D is safe and efficacious to prolong the partial clinical remission phase of T1D. Secondly, high-dose vitamin D could contribute to positive metabolic outcomes as indicated by reduced insulin requirements, excellent time in range profile, and reduced A1c. Large clinical trials of high-dose vitamin D are warranted in the general population.

## Patient perspective

Following the patient’s diagnosis with type 1 diabetes, the family reached out to our Children’s Diabetes Center at Northwell Health’s Cohen Children’s Medical Center of New York to inquire how to prolong his honeymoon phase using high-dose vitamin D based on our earlier publications in this field. We shared our protocol with them, and they, in turn, discussed this information with their endocrinologist, who was receptive to the idea of implementing adjunctive high-dose vitamin D therapy. Subsequently, the patient was started on high-dose vitamin D, given as 50,000 international units once a week for 1 month, and then once every other week for the subsequent 20 months, with his parents’ approval, supervision, and ongoing monitoring at each clinic visit by his endocrinologist. The family was very impressed with the safety profile of the adjunctive vitamin D therapy, and the fact that their child remained in the honeymoon phase throughout the duration of therapy. They adhered strictly to the protocol and were extremely pleased with the outcome as published above.

## Data Availability

The original contributions presented in the study are included in the article/supplementary material. Further inquiries can be directed to the corresponding author.
